# Upregulation of a tonoplast-localized cytochrome P450 during petal senescence in *Petunia inflata*

**DOI:** 10.1186/1471-2229-6-8

**Published:** 2006-04-13

**Authors:** Yan Xu, Hiroyuki Ishida, Daniel Reisen, Maureen R Hanson

**Affiliations:** 1 Department of Molecular Biology and Genetics, Biotechnology Building, Cornell University, Ithaca, NY 14853, USA; 2 Current address: New England BioLabs, Inc., 240 County Road, Ipswich, MA 01938, USA; 3 Laboratory of Plant Nutrition and Function, Department of Applied Plant Science, Tohoku University, Tsutsumidori-Amamiyamachi 1-1, Sendai 981–8555, Japan

## Abstract

**Background:**

Gene expression in *Petunia inflata *petals undergoes major changes following compatible pollination. Severe flower wilting occurs reproducibly within 36 hours, providing an excellent model for investigation of petal senescence and programmed cell death. Expression of a number of genes and various enzyme activities involved in the degradation and remobilization of macromolecules have been found to be upregulated during the early stages of petal senescence.

**Results:**

By performing differential display of cDNAs during *Petunia inflata *petal senescence, a highly upregulated gene encoding a cytochrome P450 was identified. Analysis of the complete cDNA sequence revealed that the predicted protein is a member of the CYP74C family (CYP74C9) and is highly similar to a tomato CYP74C allene oxide synthase (AOS) that is known to be active on 9-hydroperoxides. Cloning of the petunia genomic DNA revealed an intronless gene with a promoter region that carries signals found in stress-responsive genes and potential binding sites for Myb transcription factors. Transcripts were present at detectable levels in root and stem, but were 40 times more abundant in flowers 36 hours after pollination. Ethylene and jasmonate treatment resulted in transitory increases in expression in detached flowers. A protein fusion of the CYP74C coding region to a C-terminal GFP was found to be located in the tonoplast.

**Conclusion:**

Though oxylipins, particularly jasmonates, are known to be involved in stress responses, the role of other products of CYP74 enzymes is less well understood. The identification of a CYP74C family member as a highly upregulated gene during petal senescence suggests that additional products of fatty acid metabolism may play important roles during programmed cell death. In contrast to the chloroplast localization of AOS proteins in the CYP74A subfamily, GFP fusion data indicates that the petunia CYP74C9 enzyme is in the tonoplast. This result suggests that the highly similar CYP74C enzymes that have been identified in two other Solanaceous plants may also be associated with the vacuole, an organelle known to have a prominent role in programmed cell death.

## Background

Plant cell death occurs during the hypersensitive response [1,2], response to environmental stress [3], senescence [4], and the development of plant tissues and organs [3,5]. Among these phenomena, petal senescence is of interest both because of its importance to the horticultural industry as well as a model for programmed cell death (PCD). Petal senescence shares a hallmark feature of PCD, namely DNA fragmentation [6,7]. In contrast, an early apoptotic event common in mammalian cells, the relocation of cytochrome c from the mitochondrial membrane space into the cytosol, was not detected as a signal for wilting of petunia petal tissues [6]. Evidently some plant death processes do not necessarily require cytochrome c release as a signal. In Arabidopsis protoplasts, when death was induced by C2 ceramide, loss of mitochondrial membrane potential and cytochrome c release were observed early in the death process. However, when protoporphyrin IX was used as the induction signal, although a decrease in membrane potential occurred, cytochrome c release was not observed until after the Arabidopsis protoplasts had died [8]. In most other studies of various types of plant PCD, cytochrome c release was observed during PCD, for example, in stressed cultured cells [9,10], tapetal cells [11], proteasome-inhibited epidermal cells [12], and toxin-treated mesophyll cells [13].

Subtractive cloning and differential display have been used to identify a number of genes that are highly induced during senescence. Consistent with the profound effect of ethylene on floral senescence in ethylene-sensitive flowers, petal wilting is preceded by the up-regulation of both ACC synthase and ACC oxidase in both *Petunia *and carnation [14,15]. Most other genes up-regulated during petal senescence that have been identified so far encode enzymes involved in the degradation and remobilization of macromolecules (reviewed in [5,16]), including a thiol protease [17], acyl-CoA oxidase [18], glutathione-S-transferase [19], DNases and RNases [6,20,21], and lipoxygenases [22,23]. Other upregulated genes that have been identified have unknown functions, such as a calmodulin-binding protein [24] and a zinc-finger DNA-binding protein [25].

In the self-incompatible species *Petunia inflata*, petal senescence can be triggered reproducibly by compatible pollination, with clear wilting symptoms appearing at 36 hours after compatible pollination (HACP) [6]. This pollination-induced petal senescence not only minimizes environmental influences, but also provides an inducible system to clone and investigate up-regulated genes associated with petal cell death. Using the technique of differential display during pollination-induced petal senescence, an upregulated gene with similarity to the CYP74C subfamily of cytochrome P450s was identified. Sequence analysis indicates the predicted protein (designated CYP74C9 by the P450 nomenclature committee, identified here as PiCYP74C9) is most related to an unusual tomato allene oxide synthase that preferentially metabolizes 9-hydroperoxides [26]. While some AOS proteins have been localized to the chloroplast envelope, analysis of transgenic plants carrying PiCyP74C9 fused to GFP indicate that the *P. inflata *protein is located in the tonoplast.

## Results

### Isolation of *Psr *genes

Morphologically, programmed cell death (PCD) can be divided into two distinct stages [27], condemned/latent stage and execution stage. In the condemned stage, no obvious morphological changes are visible and the duration is quite variable. At 24 hours after compatible self-pollination (HACP), the *P. inflata *flowers appear quite normal but are condemned; at 36 hours the flowers are quite wilted [6]. To identify genes that are up-regulated during the condemned phase of petal senescence, differential display (DD) was used to compare mRNA expression profiles [28] from transcripts of young petals from flowers that just opened with those of senescing petals from flowers at 24 and 36 HACP. This comparison was to identify those genes which may be involved in early events (24 hours) during the condemned phase as well as gene involved in late events of senescence equivalent to the execution stage in apoptosis [6].

**Figure 1:**
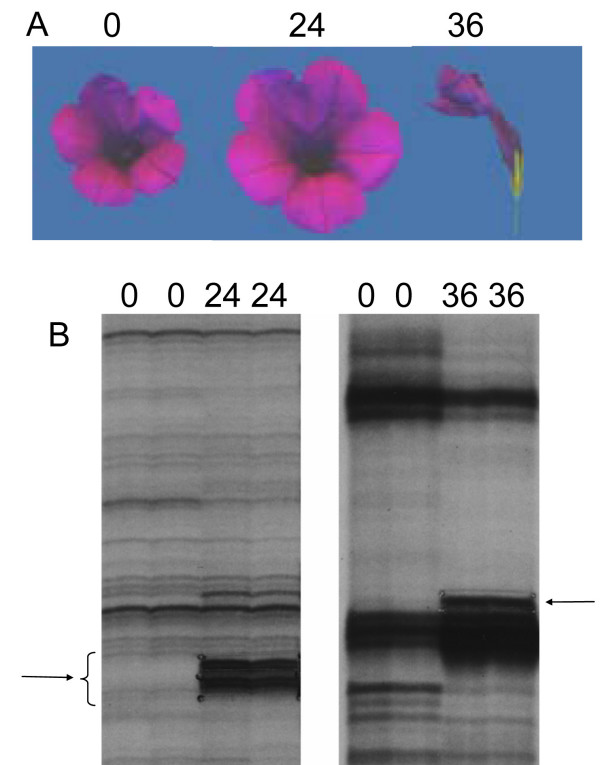
**Changes in floral morphology and RNA profiles at various hours after compatible pollination (HACP). **(A) *P. inflata *flowers at 0, 24, and 36 HACP. (B) Comparison of expressed mRNAs in petal tissues by differential display (DD). Two different primer sets were used for the DD experiments shown. Left, a DD between 0 HACP and 24 HACP. Right, a DD between 0 HACP and 36 HACP. Arrows indicate a few of the DD bands indicating upregulated transcripts during petal senescence.

Fig. 1 shows examples of DD gels comparing mRNAs of petal tissues collected at the indicated time after pollination. On the 24 HACP DD gel, most transcripts showed similar levels of expression at 0 and 24 HACP (left panel). In contrast, the steady-state level of most mRNAs at 36 HACP is reduced compared to 0 HACP (right panel). This may be due to a decreased transcription rate or an increased RNA degradation. Differential bands were cloned into TA cloning vectors and used as probes to confirm increased expression at 36 hours on Northern blots.

Using a 1:1 ratio of mRNA from petals at 24 HACP and 36 HACP, a cDNA library specific to the petal and enriched in senescence-related RNAs was constructed. The primary library was screened for the coding regions of *Psr *genes. Two independent clones contained a sequence similar to ACC oxidase, an enzyme involved in ethylene biosynthesis. Two other independent clones carried a sequence similar to a member of the plant CYP74 family, cytochrome P450s. These genes were termed petal senescence related (*psr*) genes, *Psr1 *(ACC oxidase) and *Psr2 *(encoding CYP74C9).

### Construction of full-length cDNA of *Psr2 *and characteristics of CYP74C9

**Figure 2:**
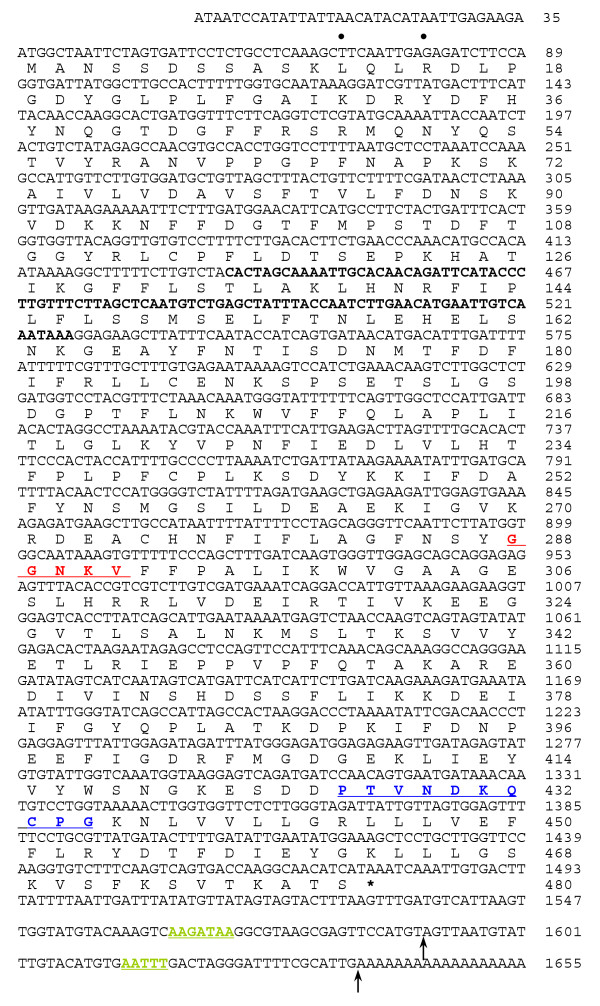
**Nucleotide sequence of *Psr2 *cDNA [Genbank: **DQ351288] **with translation of coding region. **The I-helix (red), heme-binding (blue) region, and two potential polyadenylation signals (green) are underlined. Processing sites for polyadenylation are indicated by arrows. The sequence of the longest cDNA obtained is shown; other cDNAs exhibited polyadenylation at the first arrow. The stop codon is marked with an asterisk. In addition, two nonsense codons upstream from the initiating ATG are indicated by dots. The cDNA sequence obtained by DD is boldface.

Several cDNA clones with partial coding regions were isolated for *Psr2 *by cDNA library screening. Two 3' end sequences have been identified, implying the existence of two polyadenylation sites, which are marked with arrows in Fig. 2. Surprisingly, the sequence isolated by DD is located in the 5' coding region between nucleotide 436 to 527 (Fig. 2), which is probably due to the presence of an AT rich sequence (AAATAAA). In order to obtain the 5' sequence, a 5' RACE method [29] was used and extended the 5' sequence by 72 nucleotides (Fig. 2, nucleotides 1 to 72). The longest ORF encoded by *Psr2 *is 480 amino acids, which is preceded by two in-frame nonsense codons (Fig. 2).

**Figure 3:**
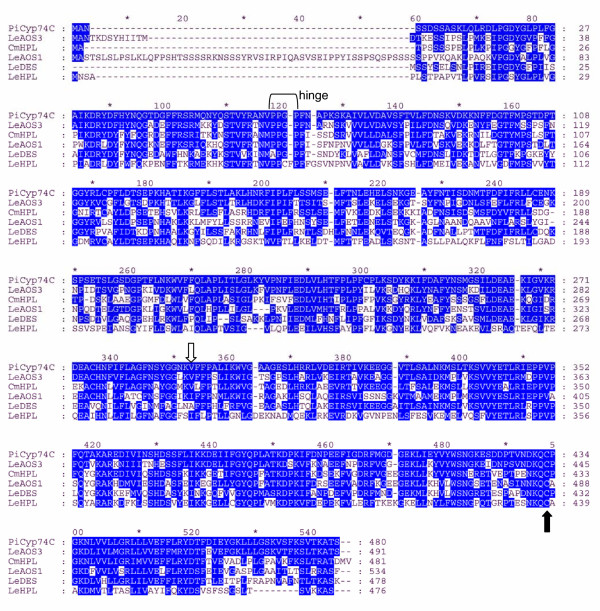
**Comparison of amino acid sequences of PiCYP74C9 with other CYP74 family members. **LeAOS3 [Genbank: z AF454634], CmHPL [Genbank: z AF081955], LeAOS1 [Genbank: AJ271093], LeDES [Genbank: AF317515], LeHPL [Genbank: z AF230372], PiCYP74C9 [Genbank: z DQ351288]. Shading indicates amino acids identical or similar to residues in PiCYP74C9. Decoration near residue 120 indicates the PPGP membrane hinge. Hollow arrow indicates conserved I/A/V found in members of the CYP74 family. The conserved cysteine present in all P450s is indicated by the solid arrow.

**Figure 4:**
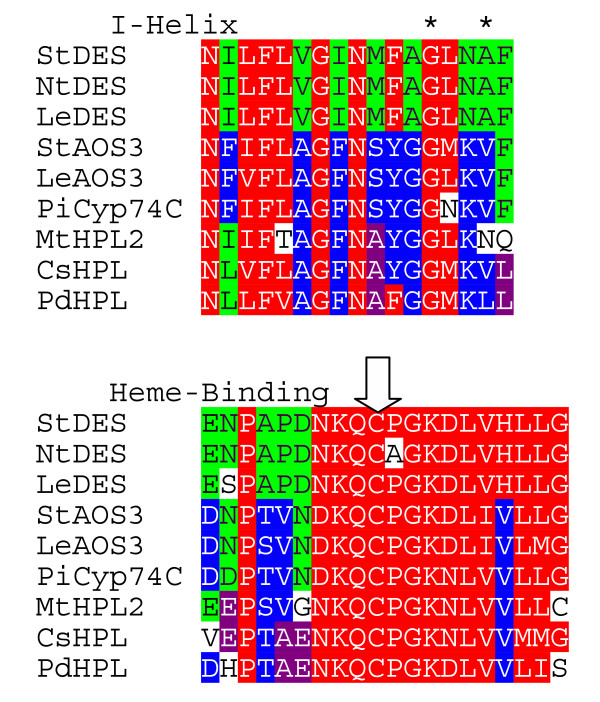
**Comparison of conserved domains of cytochrome P450s, the I-helix and heme-binding region, in the CYP74C9 subfamily. **Shading: red, conserved in most sequences; green, conserved in DES group or DES plus others; blue, conserved in AOS group or AOS plus HPL; black, conserved in HPL group. Asterisks indicate the G and I/A/V of the I-helix. Arrow indicates the conserved P450 cysteine.

The predicted protein product of *Psr2 *shows significant sequence identity to the P450 enzymes in the CYP74 subfamily (Fig. 3). Members of this family have been shown to function either as allene oxide synthase (AOS), hydroperoxide lyase (HPL), or divinyl ether synthase (DES) [30-32]. The predicted 480-amino-acid protein exhibits 68% identity and 86% similarity to a well-characterized 491 amino-acid tomato protein that has been shown to exhibit AOS activity on 9-hydroperoxides [26]. The predicted petunia protein contains a conserved PPGP tetrapeptide near the amino terminus (residue 62 to 65, Fig. 3) that is also common in P450s, a membrane hinge that is known to be important for P450 stability and catalysis [33,34]. The unique feature of the CYP74 subfamily is the oxygen-binding pocket, the I-helix. In contrast to the consensus I-helix (GxxxT) in most P450s, the threonine residue is substituted with either isoleucine, alanine, or valine (GxxxI/A/V) (Fig. 4) in AOS, HPL, and DES enzymes. The presence of GxxxV in PiCYP74C9 indicates that the protein is a peroxide dehydrase rather than an oxygenase, consistent with the known properties of this cytochrome P450 subfamily. Three members of each group within the CYP74C family and the CYP74D family are compared in two conserved regions in Fig. 4. In the I-helix region, the AOS group is more similar to the HPL group than to the DES group. In the heme binding region, the AOS group exhibits sequence similarities to both the DES and HPL groups (Fig. 4).

**Figure 5:**
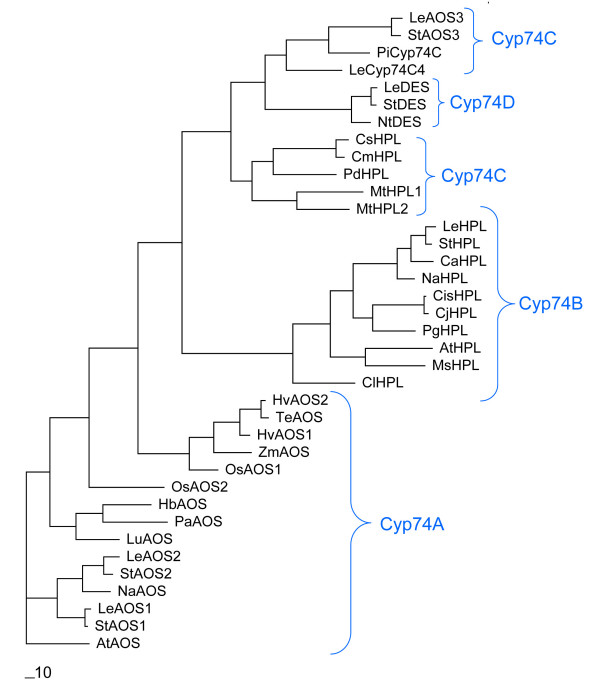
**Phylogenetic analysis of members of the cytochrome P450 74C family. **PAUP*4.0b10 [84] was used to construct a maximum parsimony tree of 37 CYP74 P-450s, with branch swapping performed by tree bisection-reconnection (TBR). AtAOS [Genbank: Y12636], AtHPL [Genbank: AF087932], CaHPL [Genbank: z U51674], CisHPL [Genbank: AY242385], CjHPL [Genbank: AB077765], ClHPL [Genbank: zAY703450, CmHPL [Genbank: z AF081955], CsHPL [Genbank: z AF229811], HbAOS [Genbank: AY514020], HvAOS1 [Genbank: AJ250864], LeAOS1 [Genbank: AJ271093], LeAOS2 [Genbank: z AF230371], LeAOS3 [AF454634], LeCYP74C4 [Genbank: AF461042], LeDES [Genbank: AF317515], LeHPL [Genbank: z AF230372], LuAOS [Genbank: z U00428], MsHPL [Genbank: z AJ249245], MtHPL [Genbank: AJ316563], MtHPL1 [Genbank: z AJ316562], MtHPL2 [Genbank: AJ316563], NaAOS [Genbank: AJ295274], NaHPL [Genbank: AJ414400], NtDES [Genbank: AF070976], PaAOS [Genbank: X78166], OsAOS1 [Genbank: AY055775], OsAOS2 [Genbank: AB116527], PdHPL [Genbank: AJ578748], PgHPL [Genbank: AF239670], PiCYP74C9 [Genbank: z DQ351288], StAOS1 [Genbank: AJ457080], StAOS2 [Genbank: AJ457081], StAOS3 [Genbank: AJ868542], StDES [Genbank: AJ309541], StHPL [Genbank: AJ310520], TeAOS [Genbank: z AY196004], ZmAOS [Genbank: AY488135]. At: Arabidopsis; Ca, pepper; Cis, orange; Cj, rough lemon; Cm, muskmelon; Cs, cucumber; Hv, barley; Le: tomato; Lu, flax; Ms, alfalfa; Mt, barrel medic; Nt, Na, tobacco; Os, rice; Pa, guayule; Pg, guava; Pi, *Petunia inflata*; St, potato.

Phylogenetic analysis (Fig. 5) is consistent with the assignment of the *Psr2 *gene product as a CYP74C family member and with its likely activity as an allene oxide synthase operating on 9-hydroperoxides. This maximum parsimony analysis organizes the proteins as expected in the CYP74A, CYP74B, and CYP74D sub-families. The CYP74C members assort into two groups, comprised of those with HPL or AOS activities, with PiCYP74C9 clearly located with the proteins with known AOS activity (Fig. 5). The tree generated by nearest neighbor analysis is quite similar, also separating the AOS and HPL CYP74C members.

### Developmental and tissue-specific expression of *Psr2*

**Figure 6:**
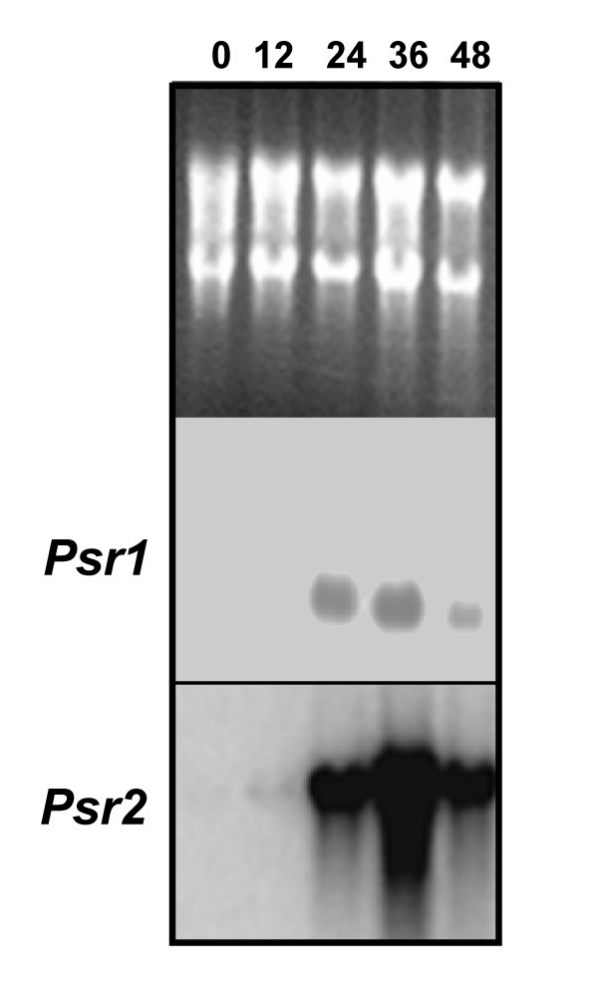
**Time course of expression of *Psr1 and Psr2 ***Total RNA (10 μg) from petals at each time point was separated on a 1.2% agarose gel containing formaldehyde, transferred to Genescreen membranes, and hybridized to probes of cDNA inserts. rRNA panel is a loading control stained with ethidium bromide. Numbers indicate HACP.

**Figure 7:**
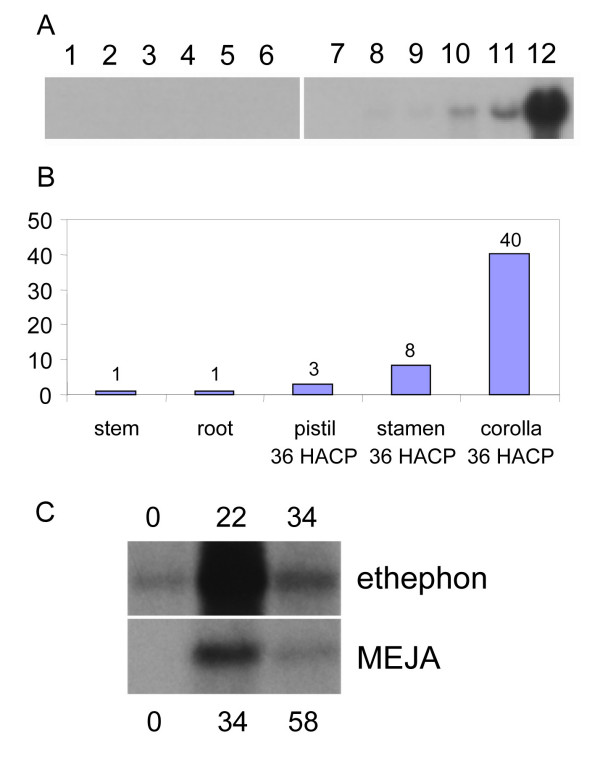
**Tissue-specificity and developmental regulation of expression of *Psr2*. **Total RNA (10 μg) from different tissues was fractionated on a 1.2% agarose gel containing formaldehyde and probed with a *Psr2 *probe. (A) Lane 1, petal (one day before opening); lane 2, corolla (just opened flowers); lane 3, corolla tube (just opened flowers); lane 4, anther (tapetum degeneration stage); lane 5, pistil (tapetum degeneration stage); lane 6, anther (one day before opening); lane 7, healthy leaf; lane 8, stem; lane 9, root; lane 10, pistil (36 HACP); lane 11, stamen (36 HACP); lane 12, petal (36 HACP). (B) Densitometry measurements of lanes 7 to 12 relative to the stem signal. (C) RNA blots from petal tissue treated with 1 mM ethephon, at 0, 22, and 34 hours after treatment or with 50 μM methyl jasmonates (MEJA) at 0, 34, and 58 hours after treatment. Shortly before 0 time, the flower petals had opened.

Analysis of the time course of *Psr2 *expression shows that *Psr2 *mRNA is not detectable on RNA blots immediately after opening but is strongly up-regulated at 24 HACP (Fig. 6). The peak expression of *Psr2 *was at 36 HACP and then declined at 48 HACP. *Psr2 *was also induced in senescing stamens and pistils at 36 HACP and senescing petals of unpollinated flowers 6 days after opening (Fig. 7), suggesting that *Psr2 *may be useful as a molecular marker of floral senescence.

Among the different tissues tested, *Psr2 *was expressed at low levels constitutively in the root and stem and at much lower levels in other young tissues, including healthy leaves and tissues from unopened flowers (Fig. 7). A densitometry measurement shows that the induced level of *Psr2 *mRNA in the pollinated petals at 36 HACP is about 40-fold higher than the constitutive level in either root or stem (Fig. 7). Since treatments with ethylene and jasmonates have been shown to accelerate petal senescence in *P. hybrida *[35,36], ethephon or MEJA was applied to detached flowers of *P. inflata*. Ethephon is a commonly used substitute for ethylene and easily converted into ethylene within plant cells [37]. In the presence of 1 mM ethephon, *P. inflata *flowers wilted within 24 hours (data not shown), whereas with simple distilled water treatment, detached flowers could last more than 6 days (data not shown). MEJA treatment seemed to promote a change in petal color rather than physical wilting (data not shown). Both ethephon treatment and MEJA treatment (Fig. 7) caused a transient increase in *Psr2 *transcripts in detached flowers.

### Characterization of Psr2 gene organization and promoter elements

**Figure 8:**
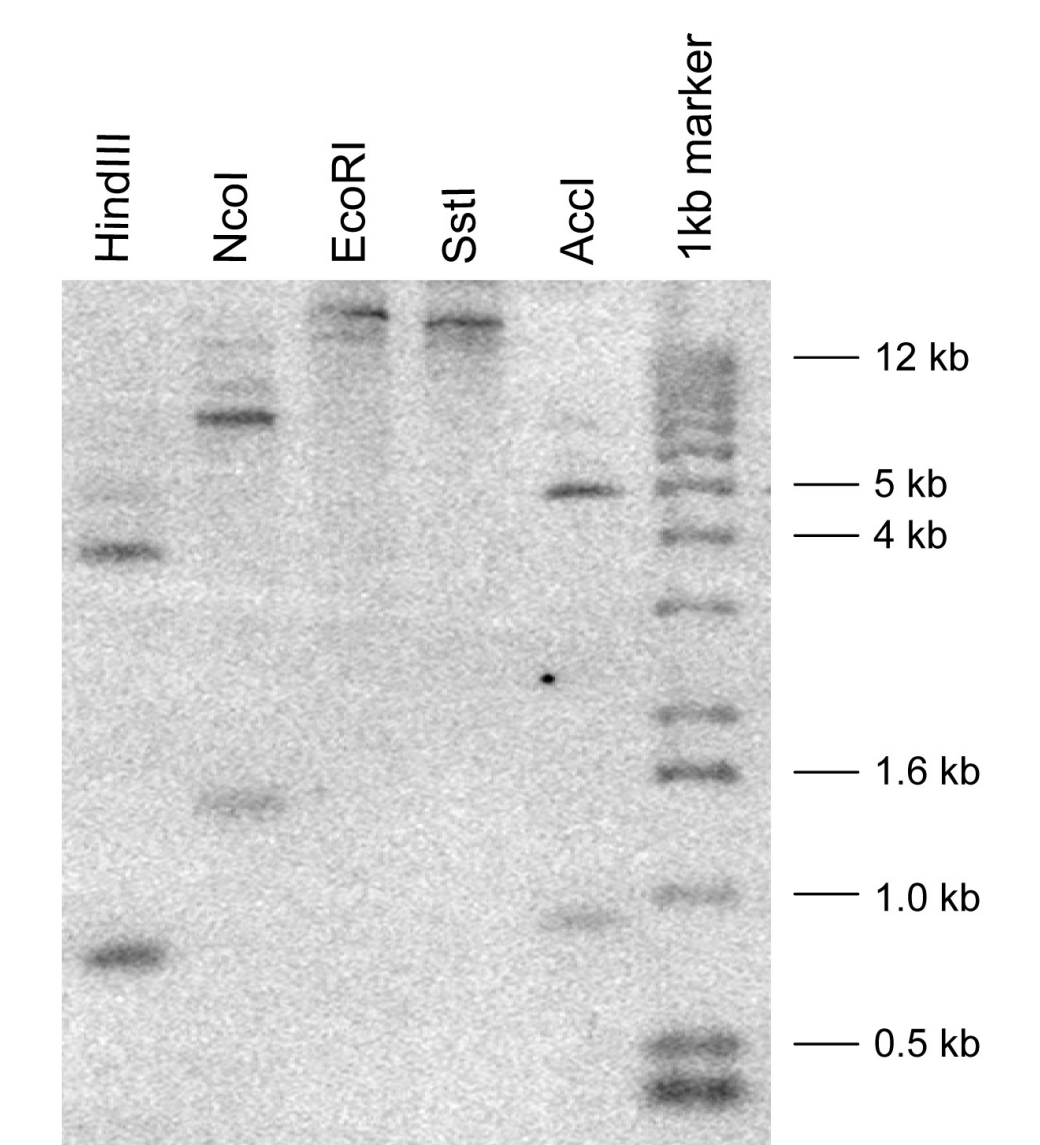
**DNA blot analysis of *Psr2*. **Equal amounts of *P. inflata *genomic DNA were digested with restriction enzymes, separated on a 1% agarose gel, transferred to a nylon membrane, and hybridized with *Psr2 *cDNA. Among the enzymes used, HindIII, NcoI, and AccI have one cleavage site in *Psr2*. EcoRI and SstI have no cleavage sites in *Psr2*.

**Figure 9:**
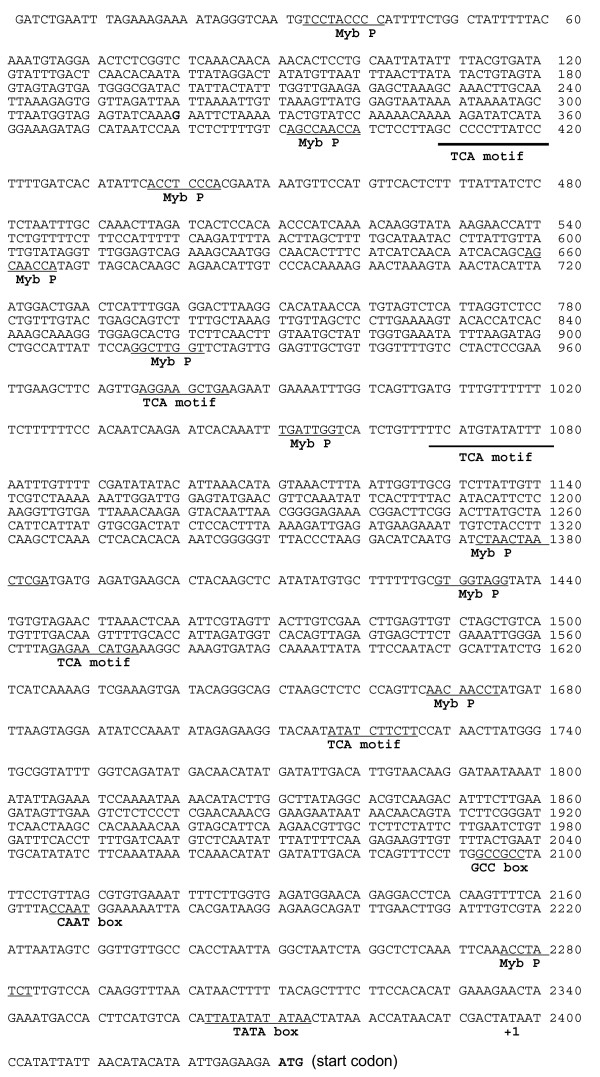
**The genomic sequence of the promoter region of *Psr2 *[Genbank: **DQ351289**]. **Putative *cis*-acting elements are underlined and labeled. The transcription start site (A) is labeled +1.

A DNA blot probed with a *Psr2 *cDNA under stringent washing conditions showed that *Psr2 *is a single copy gene (Fig. 8), as is the highly similar tomato gene encoding LeAOS3 [26]. However, additional faint bands appeared after long exposure, suggesting the presence of similar sequences in *Petunia *(data not shown). By screening a *P. inflata *genomic library [38], the promoter region and the genomic copy of *Psr2 *were isolated. In the genomic clone with the longest 5' sequence, the upstream regulatory region is 2335 bp (Fig. 9). A CCAAT box is located at -230 to -226 and a TATA box is at -33 to -22. Other *cis*-elements include a GCC box which may be ethylene responsive [39,40], three TCA motifs (common in stress-inducible genes [41]), and several potential binding sites for Myb transcription factors including maize P and *Petunia *petal epidermis-specific Myb.Ph3 [42,43]. The putative transcription start site (+1) is in agreement with both the plant consensus start sequence [44] and the sequence obtained from the 5' RACE (Fig. 9). The *P. inflata Psr2 *gene does not have introns. In the transcribed region, there is only one nucleotide difference between the *Psr2 *genomic clone and the cDNA clone we sequenced. At position 3153 there is a T in the genomic sequence and a C in the cDNA sequence; because it is in the third position of the codon, the predicted amino acid sequence is not affected.

### Cellular localization of PiCYP74C9

PiCYP74C9 lacks any defined targeting signal; analysis by a variety of web-based localization informatics programs gave inconclusive and contradictory results. To investigate the location of PiCYP74C9, the full coding region was fused in-frame to an enhanced GFP [45] and a strong promoter [46] and introduced into *Nicotiana tabacum *by Agrobacterium-mediated transformation.

**Figure 10:**
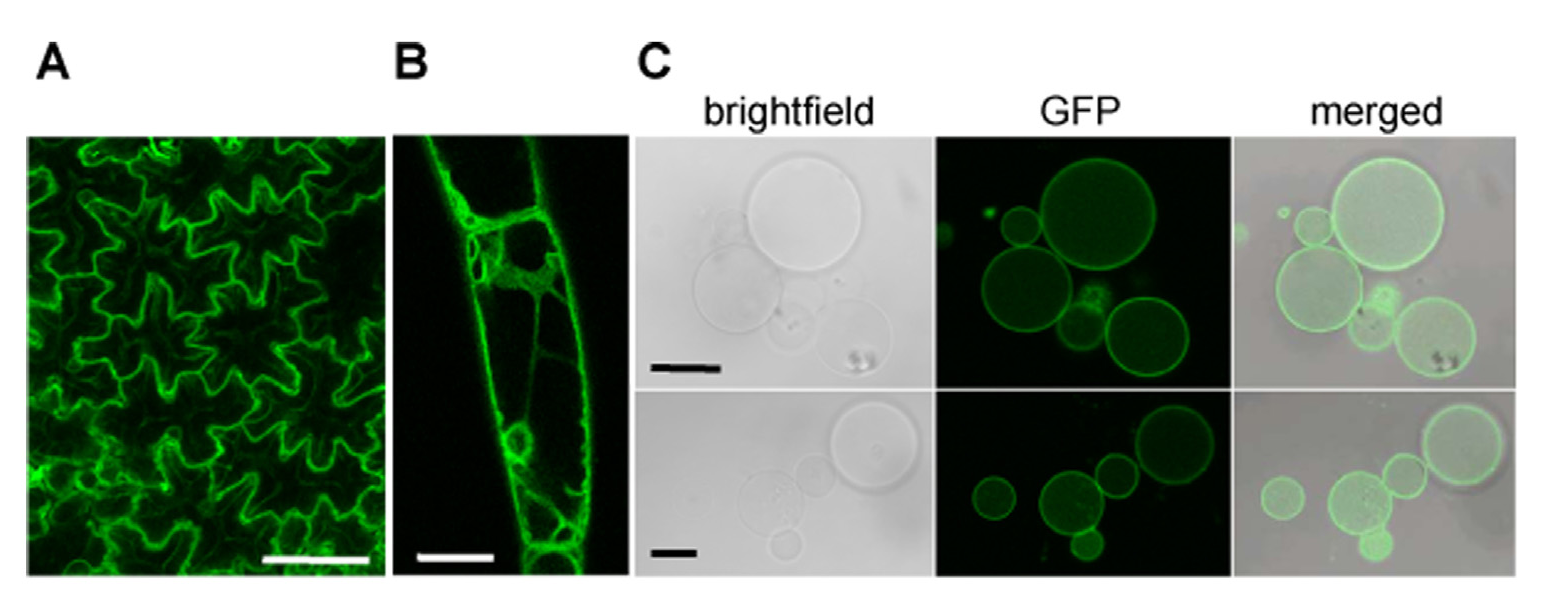
**Subcellular localization of PiCYP74C9::GFP protein assessed by microscopy. **A) epidermal cells. B, trichome cells. C, purified vacuoles. GFP signal is evident in the tonoplast. Bars = 20 μm.

A number of transgenic tobacco plants were identified that express the fusion protein at relatively high levels, according to immunoblot analysis with anti-GFP antibody (data not shown). All transgenic plants exhibited normal morphology and fertility. Microscopic analysis of transformed cells indicated that the signal was found in the tonoplast membrane (Fig. 10), as images were comparable to those obtained with a known tonoplast aquaporin/GFP fusion [47]. Images of isolated vacuoles verified the localization as in the tonoplast membrane (Fig. 10).

**Figure 11:**
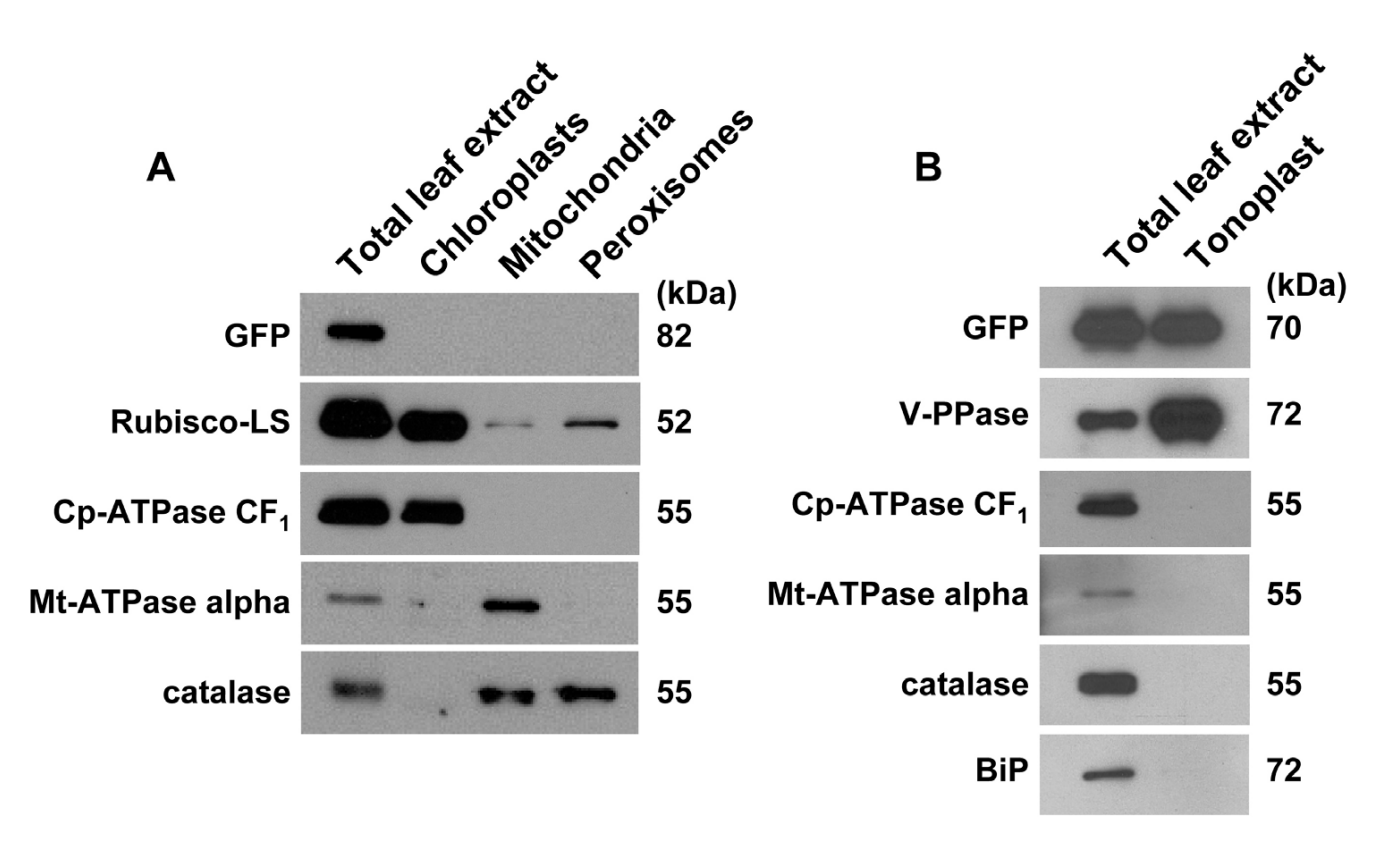
**Subcellular localization of PiCYP74C9::GFP protein by cell fractionation and immunoblot analysis. **Leaves of a transgenic tobacco plant expressing the PiCYP74C9::GFP fusion protein were fractionated into total leaf extract, chloroplasts, mitochondria, peroxisomes, and tonoplast. Proteins were separated on a 12.5% SDS-polyacrylamide gel, transferred to a nitrocellulose membrane, and probed with antibodies against GFP, the large subunit of Rubisco (Rubisco-LS), coupling factor 1 of chloroplastic ATPase (Cp-ATPase CF_1_), mitochondrial ATPase alpha subunit (Mt-ATPase alpha), catalase, vacuolar membrane proton-translocating inorganic pyrophosphatase (V-PPase), and binding protein (BiP). Protein amounts loaded were 5 μg in A or 2.5 μg in B for total leaf extracts, 3 μg for chloroplasts, 0.3 μg for mitochondria and peroxisomes, and 0.15 μg for tonoplast. Sample denaturation with SDS was done at 100°C for 3 min in A and at 50°C for 20 min in B.

Because the GFP fluorescence signal was relatively weak, despite readily detectable GFP by immunoblot analysis of total leaf extracts, we considered the possibility that non-fluorescent GFP might be located in additional subcellular organelles, especially since certain AOS enzymes are known to be located in plastids [48-50]. We fractionated cells to determine the subcellular location of the GFP fusion by immunoblot analysis. We prepared chloroplast, mitochondria, peroxisome, and tonoplast fractions and probed with an anti-GFP antibody and appropriate controls for our fractionation (Fig. 11). GFP signal was detected only in the tonoplast membrane (Fig. 11). The PiCYP74C9::GFP fusion exhibits a higher mobility when denatured at 50°C vs.100°C (Fig. 11). Proteins were treated at the lower temperature treatment (shown in Fig. 11B) because the control tonoplast marker, vacuolar pyrophosphatase, is unstable at high temperature [51].

## Discussion

The identification of macromolecules that increase in abundance during petal senescence is critical for our understanding of this process. Although a number of genes have been cloned that are highly expressed in senescing petals, the signal transduction pathways remain poorly understood. In addition to PiCYP74C9, a second highly induced known gene that we also identified during our study encodes ACC oxidase, which is involved in ethylene biosynthesis. The identification of ACC oxidase is not surprising, given that ethylene is known to be important in signaling petunia floral senescence [52,53]. The ethylene peak in *P. inflata *petal tissue starts from 18 HACP and peaks at 24 HACP [54]. This ethylene surge lags behind the up-regulation of ACC oxidase mRNA (*Psr1) *which shows up-regulation starting after 12 HACP (Fig. 6). This timing supports the theory that *de novo *synthesis of ethylene is required for the increased ethylene level.

Our previous study [6] showed that the total amount of RNA decreased about 50% at 24 HACP. Up to 36 HACP, the steady-state levels of the *Psr1 and Psr2 *transcripts continue to increase despite collapse of the floral shape and decrease in total RNA and protein contents, suggesting that *Ps*r1 and *Psr2 t*ranscripts are either continuously transcribed or protected from the large-scale degradation of RNA in the senescing petal. This indicates active regulation of petal senescence by the plant.

Both ethylene and MEJA induced expression of *Psr2 *in unpollinated petals. Jasmonates also evidently play a role in the regulation of the gene encoding LeAOS3, the CYP74C member most closely related to PiCYP74C9, as expression of LeAOS3 in roots did not occur in a tomato mutant insensitive to jasmonates [26]. LeAOS3 transcripts were found in germinating seedlings and roots but not cotyledons, mature leaves, stems, nor flower buds; evidently senescing tissue was not tested [26]. Our data suggests that it will be worthwhile to examine the expression of the genes encoding LeAOS3 and the similar StAOS3 protein in stressed tissues to find out whether all of these synthases are upregulated during programmed cell death. Likewise, it will be interesting to determine whether or not LeAOS3 and StAOS3 are targeted to the tonoplast.

**Figure 12:**
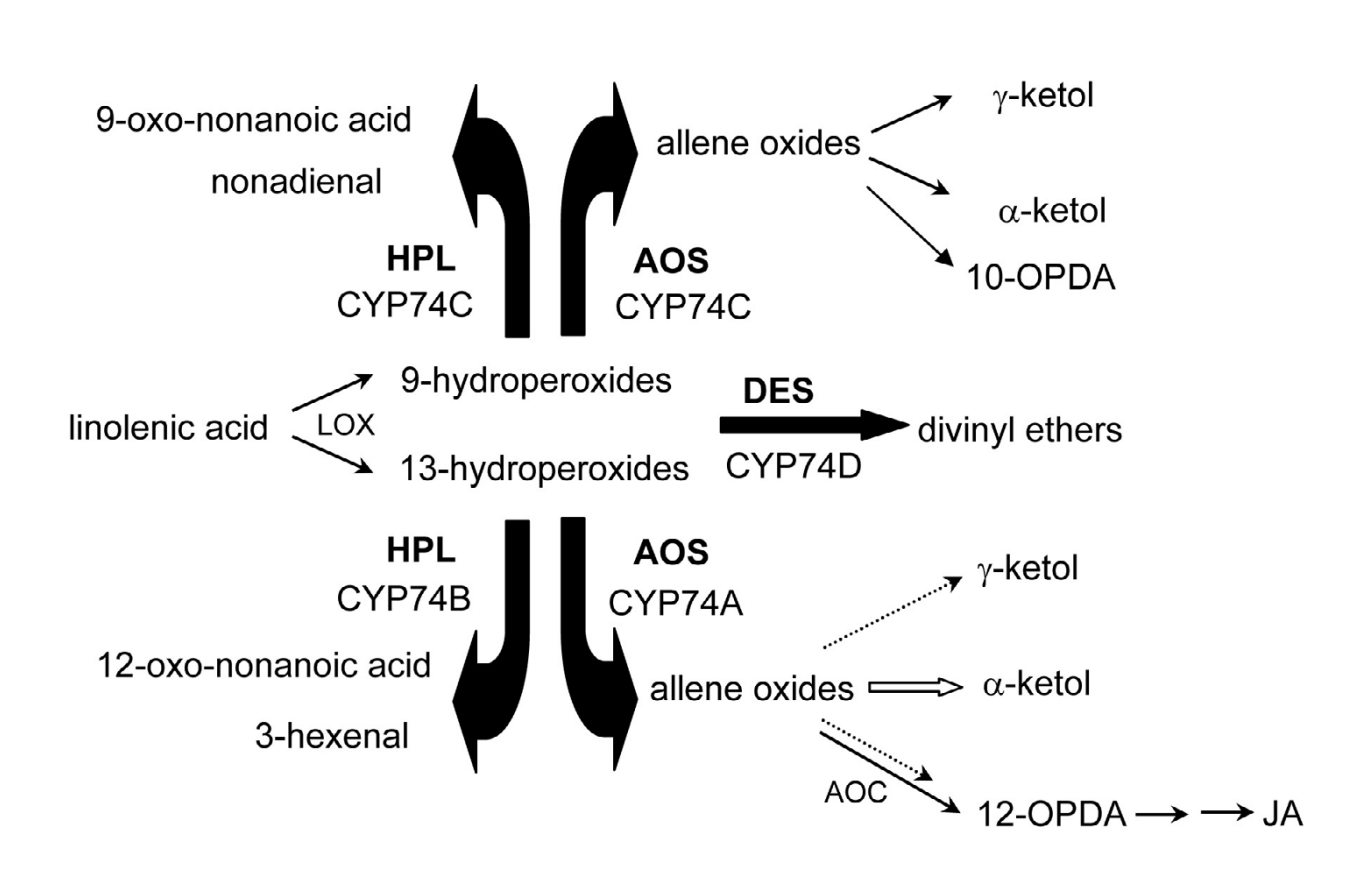
**Diagram of enzymatic activities of CYP74 family members (as reviewed in **[26,85,86]**. **LOX: lipoxygenase. AOC: allene oxide cyclase OPDA: oxo-phytodienoic acid.

P450 enzymes constitute a superfamily of enzymes that are important in the oxidative, peroxidative, and reductive metabolism of numerous endogenous compounds (reviewed in [54-56]). The CYP74 family does not require molecular oxygen, instead the oxygen is provided by the hydroperoxide substrates. There are a number of products and derivatives of the CYP74 enzymatic reactions, and some of these have well-known signaling properties while others are of unknown biological activity (Fig. 12). At present the family is divided into four sub-groups, but as more becomes known about these unusual P450s, perhaps five sub-groups will be warranted. The HPL and AOS activities on 13-hydroperoxides have been characterized in more species than the 9-hydroperoxides-preferring enzymes and clearly fall into two sub-families (Fig 5). Neither CYP74C nor CYP74D enzymes have been found in the Arabidopsis genome; indeed, at present, the AOS CYP74C enzymes are known only in Solanaceae (Fig. 5), while the HPL CYP74C enzymes have not yet been described in this family. As well as enzyme activity, future analysis may reveal whether intracellular localization also differs between sub-families and between the HPL vs. AOS CYP74C proteins.

Lipoxygenases, which act upon lipids to produce the hydroperoxide substrates utilized by CYP74 cytochrome P450s (Fig. 12), have been found in a variety of locations within the plant cell, including the vacuole [57,58]. Some CYP74 proteins have an N-terminal chloroplast transit peptide and are associated with the chloroplast. In flax, tomato, and barley, the transit sequence has been shown to be functional as targeting signal for certain AOS proteins to chloroplasts [48-50]. A tomato AOS was targeted to the outer chloroplast envelope membrane, while an HPL was targeted to the inner membrane [48]. However, other CYP74 members exist with no predictable location and await experimental determination. For example, only one of four predicted AOS genes in rice carries a putative chloroplast transit sequence [59]. Not all CYP74A members are found in the chloroplast; the guayule CYP74C AOS (PaAOS) is associated with rubber particles [60].

Our results indicate that PiCYP74C9::GFP expressed in tobacco leaves is localized in tonoplasts but not in chloroplasts, mitochondria, nor peroxisomes. We presume that PiCYP74C9 is also located in the tonoplast in petunia during petal senescence. To our knowledge, this is the first example of a CYP74 subfamily member to be localized in the tonoplast. Both N-terminal [61] and C-terminal [47] GFP fusions with known tonoplast proteins have resulted in GFP labeling of the tonoplast. None of the computer programs we tested (Predotar, TargetP, Psort) predicted PiCYP74C9 to be located in either plastids, mitochondria, or the secretory pathway.

The tonoplast localization of PiCYP74C9 is intriguing given recent information about the important role of the vacuole in programmed cell death. While it has been known for some time that caspase activity is involved in both developmental and hypersensitive response cell death, only recently has the vacuolar processing enzyme been shown to exhibit caspase activity that is important for the execution phase of several types of PCD [62-64]. During senescence, cell contents can be recycled by digestion in vacuoles in the process of autophagy [65]. In some types of developmental programmed cell death, including petal senescence, the final stages coincide with ultrastructural changes and permeabilization of the tonoplast and other membranes (reviewed in [5,16,66]). Confirmation of the allene oxide synthase activity of PiCYP74C9 on 9-hydroperoxides and identification of the products may give insights into the role of this protein in petal senescence. Future analyses of the PiCYP74C9 protein should also reveal the significance of its compartmentation in the tonoplast.

## Conclusion

By the technique of differential display, we have identified a cytochrome P450 that is expressed at a level 40 times greater in senescing petals than in vegetative tissue. Upregulation occurs in response to compatible pollination, ethylene treatment, or jasmonate treatment. The petunia gene encodes a protein highly similar to a tomato CYP74C protein known to exhibit allene oxide synthase activity, preferentially on 9-hydroperoxides. Both a complete cDNA and genomic sequence of this single-copy gene have been obtained The promoter region of the petunia gene exhibits several motifs found in stress-responsive genes as well as binding sites for a petunia transcription factor. A C-terminal GFP fusion protein was located in the tonoplast, a compartment where CYP74 members have not previously been detected. Phylogenetic analysis indicates that the CYP74C subfamily may warrant future division into two groups, as more information becomes available about AOS and HPL enzymes acting on 9-hydroperoxides.

## Methods

### Plant materials, growth, and pollination

Two different *P. inflata *populations bearing different S alleles can be used to pollinate each other. A line termed *P. inflata-1 *was derived from seed originally received from Ken Sink (Michigan State). The other population, termed P-S-14, was provided by D. Maizonnier (Dijon, France), who obtained it from a South American source. Plants were grown at 23°C under 16 hr daylight and 8 hr darkness. The compatibility of P-S-14 pollen on *P. inflata-1 *was confirmed by seed production, while self-pollination of *P. inflata-1 *flowers did not result in seed set. Pollen from P-S-14 was used to pollinate *P. inflata-1 *on the day of flower opening. At pollination, the five stamens were removed from *P. inflata-1 *flowers to reveal the stigma and to avoid incompatible pollination.

Transgenic tobacco cv. Petit Havana plants containing the PiCYP74C9::GFP fusion that were used for cellular fractionation were grown in a growth room at 23°C under 16 hr light and 8 hr darkness for about one month before organelle isolation. For fluorescence microscopy observations, transgenic tobacco plants containing the PiCYP74C9::GFP fusion were grown in a greenhouse under natural illumination. Both GFP genes were under the control of the 35S promoter with AMV translation enhancer [46] in the PGTPV-Kan vector [67]. Transgenic tobacco plants were produced as described by Kwok and Hanson [68].

### Chemical treatments of detached flowers

Flowers were cut at the pedicel the day of opening. Twelve flowers were used in each treatment with pedicel placed in the chemical solution held in a 24-well tissue culture plate (Northeast Container Corporation, Dover, NH). The plate was left in the greenhouse with 16 hr light and 8 hr darkness. The stock solution for ethephon (Sigma Chemical, St. Louis, MO) was 1 mM in ddH_2_O. The stock solution for MEJA (Bedoukian Research Inc., Danbury, CT) was 100 mM in 95% ethanol. Stock solutions were kept at -80°C. The working solution was diluted from the stock solution in ddH_2_O before use.

### RNA isolation and RNA blot analysis

Total RNA was extracted with TRIZOL reagent according to the manufacturer's instruction (GIBCO BRL). RNA amounts were determined by OD_260_. RNA electrophoresis (10 μg total RNA per lane) was carried out in formaldehyde denaturing gels as described (Sambrook et al. 1989), but the concentration of formaldehyde in the gel and running buffer was reduced to 0.7 M. RNA was transferred to Genescreen membrane (NEN Research Products, Boston, MA) in 20xSSC and hybridized at 65°C overnight in Church buffer (250 mM NaPO4, pH 7.2, 7% SDS, 1% BSA, 1 mM EDTA). Probe labeling was done with the random priming labeling kit DECAprimeII (Ambion, Austin, TX). After overnight hybridization, filters were washed twice with 0.2xSSC/0.1%SDS at 65°C.

### Differential display

Total RNA was extracted from 10 petals at 0, 24, and 36 HACP using TRIZOL reagent with the following modification from standard protocol. Before isopropanol precipitation, two rounds of ether extraction were added. After isopropanol precipitation, RNA was dissolved in ddH_2_O and precipitated in the presence of 2 mM LiCl at 4°C overnight. RNA concentration was determined by A_260_. For differential display, 1.5 μg total RNA from 0 HACP was compared with that of 24 or 36 HACP following the instructions for differential display provided by GenHunter (Brookline, MA). Reagents used in reverse transcription were from GIBCO BRL and the PCR buffer was from GenHunter. The primers for differential display were a generous gift from Dr. Mikhail Nasrallah.

### DNA isolation and blot analysis

Genomic DNA was isolated according to a modified CTAB method [69]. For DNA blot analysis, equal amounts of genomic DNA were digested with restriction enzymes, separated on a 1% agarose gel, and transferred to Hybond N^+ ^(Amersham Pharmacia). The hybridization was done as described for RNA blot analysis.

### cDNA library construction and screening

Total RNA isolation was performed following a phenol/SDS method [70] with modifications. Five grams of petals from 24 HACP or 36 HACP were ground in liquid N_2 _and further ground after adding 40 ml NES buffer (100 mM NaCl, 5 mM EDTA, 1% SDS) and 20 ml sodium acetate-buffered phenol (pH 4.0). The mixture was then homogenized with a Polytron (Brinkmann Instruments Inc., Westbury, NY) for 2 min. Messenger RNA was purified through an oligo(dT)-cellulose column (type 7) following the manufacturer's instruction (Amersham Pharmacia). Handling of the cDNA library, including construction, titering, screening, and *in vivo *excision, followed the manufacturer's instructions. The ZAP Express cDNA synthesis kit and ZAP Express cDNA Gigapack III gold cloning kit (Stratagene, La Jolla, CA) were used for the cDNA library construction. Starting with 5 μg mRNA from senescing petals (2.5 μg from 24 HACP and 2.5 μg from 36 HACP), a primary cDNA library with about 540,000 recombinant phage was obtained. About 25,000 primary phage were screened to clone the gene.

### Cellular fractionation

Tobacco leaves were homogenized in Hepes-NaOH (pH 7.5) containing 14 mM 2-mercaptoethanol and protease inhibitor cocktail (Complete Mini, Roche) in a chilled mortar and pestle. The homogenate was mixed with an equal volume of SDS sample buffer consisting of 200 mM Tris-HCl (pH 8.5), 2% (w/v) SDS, 0.7 M 2-mercaptoethanol, and 20% (v/v) glycerol and then it was boiled for 3 min or heated at 50°C for 20 min when used in comparison with tonoplast vesicles. Following microcentrifugation, the supernatant was taken for use as the whole leaf extract.

For the isolation of organelles, leaves were cut into small slices (approximately 1 mm× 10 mm) with a razor blade and homogenized in a Polytron for 2 sec five to seven times with 5 ml of a homogenizing buffer per gram fresh weight of leaves.

Chloroplasts were isolated from 30 g of leaves essential as described previously [71] except that the extraction and Percoll gradient buffer contained 2 mM EDTA instead of 10 mM EDTA. Intact chloroplasts were collected from a green layer near the bottom of Percoll gradients, suspended in 50 mM Hepes-NaOH (pH 8.0) containing 0.3 M mannitol and 2 mM EDTA, and centrifuged at 3,000 g for 40 s. The resulting pellet was used as the purified chloroplast fraction.

Mitochondria were isolated from 60 g of leaves using Percoll gradient centrifugation with 0–5% (w/v) PVP-40 preformed gradient based on the method described by Day et al[72] with slight modifications as follows. Leaves were homogenized with a grinding buffer consisting of 25 mM Mops-KOH (pH 7.8) containing 0.4 M mannitol, 10 mM Tricine, 8 mM cysteine, 1 mM EGTA, 1% (w/v) PVP-40, and 0.1% (w/v) BSA. The homogenate was passed through eight layers cheesecloth and centrifuged at 1,000 g for 5 min. The supernatant was centrifuged again at 12,000 g for 15 min. The pellet was washed with 25 mM Mops-KOH (pH 7.2) containing 0.4 M mannitol and 1 mM EGTA and was suspended in the same buffer and layered on top of a solution of 28% (v/v) Percoll in 25 mM Mops-KOH (pH 7.2) containing 0.4 M mannitol and 0.1% (w/v) BSA with 0–5% (w/v) PVP-40 preformed gradient. After centrifugation at 40,000 g for 45 min, white band near the bottom of the tube was collected, suspended in 25 mM Mops-KOH (pH 7.2) containing 0.4 M mannitol and 1 mM EGTA, and centrifuged at 12,000 g for 15 min. The resulting pellet was used as the purified mitochondrial fraction.

Peroxisomes were isolated from 30 g of leaves based on the method described by Fukao et al. [73] as follows. Leaves were homogenized with a grinding buffer consisting of 20 mM pyrophosphate-HCl (pH 7.5) containing 0.3 M mannitol and 1 mM EDTA. The homogenate was passed through four layers of cheesecloth. The residue was homogenized with another grinding buffer again in a similar manner. The filtrates were combined and were centrifuged at 1,500 g for 10 min. The supernatant was centrifuged again at 10,000 g for 20 min. The pellet was washed with a grinding buffer, and was suspended in 4 ml of 10 mM Hepes-KOH (pH 7.2) containing 0.3 M mannitol and 1 mM EDTA and was subjected to centrifugation in Percoll. The suspension was layered on top of 5 ml of a 60% (v/v) and 30 ml of a 28% (v/v) solution of Percoll in 10 mM Hepes-KOH (pH 7.2) containing 1 mM EDTA and 0.3 M raffinose and was centrifuged at 40,000 g for 30 min without deceleration. After centrifugation, 1 ml fractions were collected by a fraction collector. The 6th fraction from the bottom, which had the highest catalase content judging from immunoblot analysis, was used as the purified peroxisome fraction.

Vacuoles were isolated from leaf protoplasts as follows. Leaves (15 g) were cut into small slices as described above and digested with an incubation medium conainting 1.5% Cellulase Onozuka RS, 0.4% Macerozyme R-10, 10 mM Mes-NaOH (pH 5.6), 8 mM CaCl_2 _and 0.7 M mannitol for 3 h at 30°C in darkness. After digestion, released protoplasts were passed through a 150-μm nylon mesh and washed with 0.7 M mannitol for three times. Protoplasts were treated with DEAE-dextran solution for the lysis and released vacuoles were separated by the discontinuous Ficoll gradient centrifugation as previously described by Asami et al. [74].

Tonoplast vesicles were isolated from 20 g of leaves based on the method described by Maeshima and Yoshida [75] as follows. Leaves were homogenized with a grinding buffer consisting of 50 mM Tris-acetate (pH 7.5) containing 0.25 M sorbitol, 2 mM EGTA, 1% (v/v) PVP-40, 2 mM DTT and 0.5 mM PMSF. The homogenate was passed through four layers of cheesecloth. The filtrates were centrifuged at 3,600 g for 10 min. The supernatant was centrifuged again at 120,000 g for 30 min. The pellet was suspended in 15 ml of 20 mM Tris-acetate (pH 7.5) containing 0.5 M sucrose, 1 mM EGTA, 2 mM DTT and 2 mM MgCl_2 _and poured into a centrifugation tube. The suspension was overlayed with 15 ml of 20 mM Tris-acetate (pH 7.5) containing 0.25 M sorbitol, 1 mM EGTA, 2 mM DTT and 2 mM MgCl_2_. After centrifugation at 120,000 g for 30 min in a Beckman 70 Ti rotor, the interference portion was collected and diluted in a three-times volume of 20 mM Tris-acetate (pH 7.5) containing 0.25 M sorbitol, 1 mM EGTA, 2 mM DTT and 2 mM MgCl_2_. The suspension was then centrifuged at 120,000 g for 30 min and the resulting pellet was used as the tonoplast preparation.

### Immunoblot analysis

Purified organelles, except tonoplast vesicles, were mixed with an equal volume of SDS sample buffer as above and then boiled for 3 min. Tonoplast vesicles were denatured at 50°C for 20 min. Protein concentration was determined by RC DC Protein Assay (Bio-Rad) based on Lowry method [76] according to the manufacturer's instruction. BSA was used as standard. Protein amounts loaded on 12.5% (w/v) SDS-PAGE gels were 5 μg for whole leaf extract, 3 μg for chloroplasts, 0.3 μg for mitochondria and peroxisomes, and 0.15 μg for tonoplasts. Immunoblot analysis was performed by the method of Towbin et al. [77].

Antibodies used were rabbit anti-GFP antibodies (1:5,000, Molecular Probes), affinity-purified anti-large subunit of Rubisco antibodies (1:20,000; [78]) from rabbit anti-rice whole Rubisco antibodies [79] and rabbit anti-rice coupling factor 1 of chloroplastic ATPase antibodies (1:5,000; [80]), both gifts of Dr. Amane Makino; a mouse anti-maize mitochondrial ATPase alpha subunit antibody (1:200; [81]); a mouse anti-tobacco catalase monoclonal antibody purchased from Princeton University Molecular Biology Department Monoclonal Antibody Facility (1:500; [82]); anti-peptide antibody for vacuolar pyrophosphatase corresponding to the sequences HKAAVIGDTIGDPLK (putative loop XII, 1:50,000), a gift of Dr. Philip Rea [51]; and anti-binding protein, a gift of Dr. Eliot Herman (1:50,000, [83]).

### Microscopy

Laser-scanning confocal and differential interference contrast microscopy was performed with a Leica TCS-SP2 confocal scanning head mounted on a Leica DMRE-7 (SDK) upright microscope equipped with a 100×HCX PL APO oil immersion objective (NA = 1.40; Leica Microsysytems Inc., Bannockburn, IL, USA). GFP was excited with the 488 nm line of a 4-line Argon ion laser and emission of GFP was detected between 500 and 581 nm.

## Authors' contributions

YX cloned *Psr *cDNAs and *Psr2 *genomic DNA, performed expression analyses, and regenerated transgenic plants carrying the PiCYP74C9::GFP fusion. HI fractionated cells of transgenic plants and carried out immunoblot analysis. DR made the microscopic observations. Both YX and MRH performed bioinformatic analyses and wrote the manuscript. MRH coordinated the project and edited the final manuscript, which was approved by all authors.
